# Microclimatic and Anthropogenic Drivers of Insect Biodiversity in Rubber-Based Agroforestry Systems

**DOI:** 10.3390/insects17020195

**Published:** 2026-02-12

**Authors:** Jian Pan, Mo Yang, Yewei Wang, Tianliang Xu, Jun Tao, Beibei Zhang

**Affiliations:** 1 School of Tropical Agriculture and Forestry, Hainan University, Danzhou 571737, China; pianjian529@126.com (J.P.); 23220954000055@hainanu.edu.cn (M.Y.);; 2 Rubber Research Institute, Chinese Academy of Tropical Agricultural Sciences, Haikou 571101, China

**Keywords:** rubber plantations, agroforestry systems, insect diversity, environmental drivers

## Abstract

Rubber (*Hevea brasiliensis*) plantations constitute the largest artificial ecological forest systems in tropical regions of China, while long-term monoculture has significantly reduced biodiversity, particularly among insect communities. Rubber-based agroforestry systems are widely recognized as a promising approach to improving ecosystem functionality. However, the mechanisms by which different intercropping patterns affect insect community dynamics remain poorly understood. This study systematically evaluated the effects of eight rubber-based agroforestry systems on insect community diversity, functional group composition, and associated environmental drivers. Using rubber monoculture as a control, seven rubber-based agroforestry systems were investigated from April 2024 to March 2025. A total of 94,483 insect individuals belonging to 16 orders, 222 families, and 1560 species were recorded. The results indicate that the rubber–fig (*Ficus hirta*) and rubber–banana (*Musa nana*) agroforestry systems supported higher insect richness, diversity, and community stability than other systems, while the more complex rubber–coconut (*Cocos nucifera*)–fig (*Ficus hirta*) system exhibited a relatively lower value. Functionally, herbivores dominated the rubber monoculture system. The moderately grazed rubber–forage grass (*Brachiaria eruciformis*)–black goat agroforestry system promoted predators and detritivores, whereas the rubber–konjak (*Amorphophallus bulbifer*) agroforestry system attracted more omnivores. The permutational multivariate analysis of variance revealed that insect species composition was primarily negatively driven by canopy cover (R^2^ = 14.65%) and management intensity (R^2^ = 11.54%). The ecological benefits of rubber-based agroforestry systems depend not only on crop species diversity but also on vegetation structural complexity and management practices. It is recommended to promote the rubber–banana and rubber–fig agroforestry systems as optimized models and to enhance insect-mediated ecosystem services by maintaining understory vegetation structure, regulating canopy cover, and implementing low-intervention management practices.

## Simple Summary

Insect diversity is vital for ecosystem health but declines significantly in tropical monoculture rubber plantations. Agroforestry systems that combine rubber with other plants offer a promising alternative, yet the influence of varying planting configurations on insect communities remains poorly understood. This year-long study conducted in Hainan, China, compared insect assemblages across multiple rubber-based agroforestry systems and a conventional rubber monoculture. The results indicate that the type of agroforestry system significantly influences insect community composition. Systems like the rubber–fig (*Ficus hirta*) and rubber–banana (*Musa nana*) supported higher levels of insect diversity and stability, while a more complex rubber–coconut (*Cocos nucifera*)–fig (*Ficus hirta*) system exhibited relatively lower performance. Furthermore, different systems also favored distinct functional groups: monoculture attracted more herbivores, while other diversified systems supported more predators, detritivores, and omnivores. Canopy cover and management intensity were identified as the primary negative drivers of insect diversity, whereas flowering intensity and vegetation cover emerged as the main positive factors. We recommend promoting the rubber–banana and rubber–fig systems as optimized models and enhancing insect ecosystem services through maintaining understory vegetation structure, regulating canopy cover, and adopting low-intervention management practices.

## 1. Introduction

Insects, the most species-rich group of organisms on Earth, play a crucial role in maintaining biodiversity and sustaining the functionality of terrestrial ecosystems. They contribute to ecosystem stability and health through pollination, pest regulation, decomposition, and energy transfer [1,2,3,4,5]. Due to their high sensitivity to environmental changes, insect community composition and diversity serve as effective indicators of habitat quality and overall ecosystem health, providing valuable insights into how ecosystems respond to environmental perturbations [6]. Therefore, understanding the drivers that shape insect diversity is fundamental to elucidating the structure, function, and maintenance mechanisms of ecosystems.

Agricultural practices significantly shape insect communities by altering habitat conditions. Different planting patterns modify forest microclimate (e.g., temperature, humidity, and light availability), influence food availability (e.g., through flowering plants), and alter the intensity of human disturbance by changing vegetation structure complexity, thereby affecting species composition, abundance, and functional group organization [7,8,9]. Complex intercropping systems improve insect habitat quality by stabilizing microclimatic conditions and increasing resource diversity, which in turn shapes community structure [10]. In contrast, intensive management practices such as frequent weeding simplify habitats and diminish resources for beneficial insects [11]. These results underscore microclimate regulation, resource availability, and human management intensity as key environmental drivers mediating the effects of agricultural practices on insect communities. However, the relative importance of these drivers across diverse agroforestry systems remains poorly understood.

*Hevea brasiliensis* is a major economic tree species cultivated in tropical regions for its natural latex, an essential industrial raw material widely utilized in national defense, transportation, and medical applications [12]. In China, *H. brasiliensis* is predominantly grown in tropical areas such as Hainan and Xishuangbanna, Yunnan Province, with Hainan Island accounting for approximately half of the national plantation area [13]. However, extensive monoculture rubber plantations have led to significant biodiversity loss and degradation of ecosystem functions [14,15,16,17]. To balance economic productivity with ecological sustainability, rubber-based agroforestry systems have been promoted as viable alternatives [18,19,20,21,22,23,24]. In the initial phase of enhancing the economic returns of rubber plantations, researchers primarily focused on identifying crop species suitable for intercropping within rubber-based agroforestry systems. However, over the long-term management cycle of rubber plantations, light limitation due to canopy shading from mature rubber trees emerged as a dominant constraint impeding the development of rubber-based intercropping. To address these limitations, Huang et al., following 17 years of research, proposed a double-row planting system. This optimized planting system facilitates long-term intercropping of rubber trees with a broader range of companion crops, thereby sustaining enhanced economic benefits without compromising rubber yield [22]. While stable economic gains are now attainable in rubber plantations, increasing emphasis is being placed on ecological benefits by researchers. Evidence indicates that clonal rubber agroforestry represents a viable compromise, delivering modest improvements in biodiversity—particularly for certain taxa such as butterflies, birds, and reptiles—without requiring the expansion of cultivated land or compromising rubber yield in Thailand [23]. These systems transform monocultures into multi-layered crop configurations that mimic natural forest structure, enhancing vegetation heterogeneity and improving habitats for local insect communities [25]. Despite increasing interest, prior studies have primarily compared monoculture systems with agroforestry systems, lacking a systematic evaluation of how different intercropping patterns influence insect assemblages and their underlying drivers—such as vegetation cover and management intensity [26,27,28]. This knowledge gap limits the understanding of the ecological value of rubber agroforestry and hinders the development of sustainable management strategies.

To address this, we examined eight representative rubber planting patterns on Hainan Island by employing a comprehensive sampling approach that combined line transects, light traps, and Malaise traps across both the rainy and dry seasons. Concurrently, we recorded environmental data, including understory vegetation structure, human management practices, and climatic variables. Through detailed analysis of community composition, functional group dynamics, diversity indices, and environmental responses, our study aimed to: (1) characterize insect community structure and diversity across rubber-based agroforestry systems; (2) evaluate functional group responses to different intercropping patterns; and (3) identify key microclimatic, vegetation, and anthropogenic drivers shaping insect communities. These findings would provide a scientific foundation for biodiversity conservation and ecologically sustainable rubber forest management in tropical regions.

## 2. Materials and Methods

### 2.1. Study Area and Plot Design

The study was conducted at the experimental farm of the Chinese Academy of Tropical Agricultural Sciences in Danzhou City, Hainan Province, China (19°53′–19°56′ N, 109°47′–109°50′ E), covering approximately 1520 hectares. This farm serves as the only comprehensive demonstration base on Hainan Island dedicated to rubber-based agroforestry systems for understory resource utilization, providing a platform for research on sustainable rubber plantation management. Located on the northern edge of the tropics, the area experiences a typical tropical monsoon climate with abundant light and heat resources. The mean annual temperature is 23.5 °C, and the average annual rainfall is 1815 mm, with distinct dry and rainy seasons.

**Figure 1:**
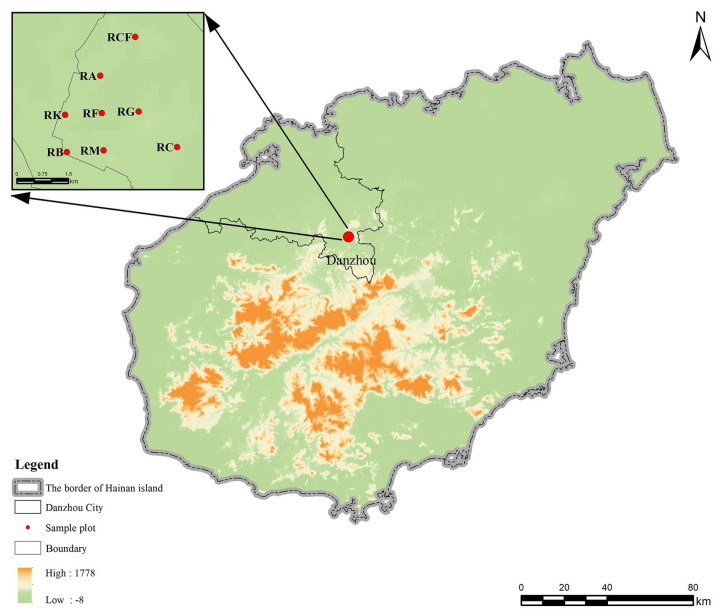
Distribution of surveyed plots within the study area and location on Hainan Island. RCF: Rubber–coconut–fig agroforestry system; RA: Rubber–*Alpinia* agroforestry system; RK: Rubber–konjak agroforestry system; RF: Rubber–fig agroforestry system; RG: Rubber–forage grass–black goat agroforestry system; RB: Rubber–banana agroforestry system; RC: Rubber–coconut agroforestry system; RM: Rubber monoculture system.

**Figure 2:**
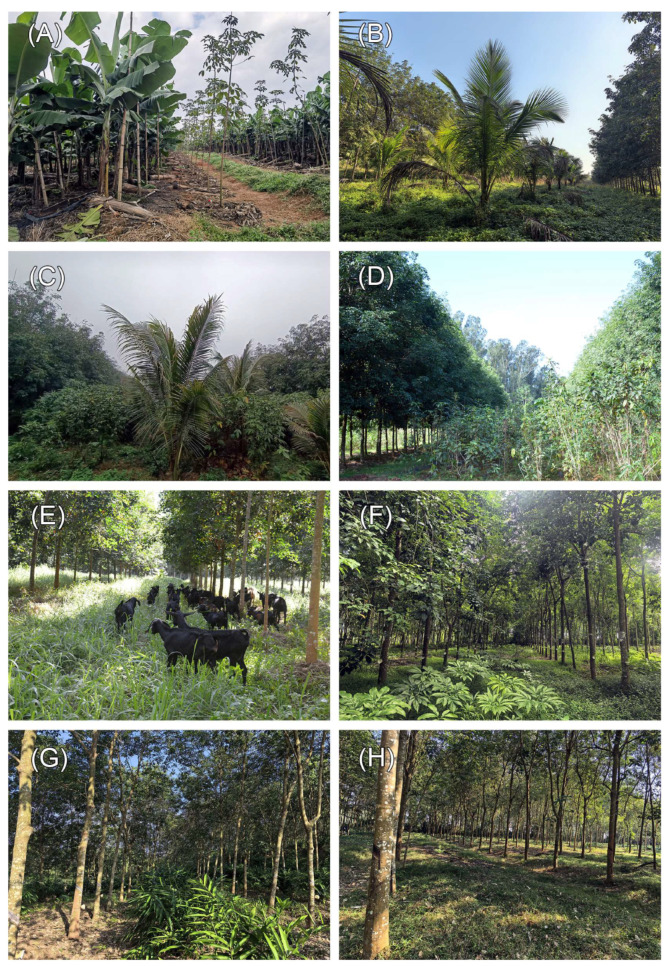
Photographic documentation of eight rubber-based agroforestry system habitats. (**A**) Rubber–banana agroforestry system; (**B**) Rubber–coconut agroforestry system; (**C**) Rubber–coconut–fig agroforestry system; (**D**) Rubber–fig agroforestry system; (**E**) Rubber–forage grass–black goat agroforestry system; (**F**) Rubber–konjak agroforestry system; (**G**) Rubber–*Alpinia* agroforestry system; (**H**) Rubber monoculture system.

The landscape is predominantly characterized by intensively managed rubber plantations, with interspersed economic crops establishing agroforestry systems in certain sections. Eight representative rubber-based agroforestry systems were selected as experimental plots (Figure 1), based on the official Catalog of “Ten Major Development Models for Understory Economy in Natural Rubber Plantations” circular from the Department of Agriculture and Rural Affairs of Hainan Province and supplemented by prevailing local practices [29]. Each plot covered > 10,000 m^2^, with inter-plot distances < 4 km and a topographic slope < 5°, surrounded by monoculture rubber stands. To minimize edge effects, all plots were established at a minimum distance of 30 m from the boundaries of the agroforestry habitat. Detailed characteristics of the selected agroforestry systems, including planting patterns, canopy cover, and management practices, are summarized in Table 1 and Figure 2. Based on canopy cover, rubber-based agroforestry systems are classified into three distinct stages: low canopy cover (0–20%, characterized by high light intensity), moderate canopy cover (30–60%, characterized by moderate light intensity), and high canopy cover (75–95%, characterized by low light intensity). The selection of intercropping species at each growth stage is determined by their specific light requirements. Shade-intolerant or high-light-demanding species are generally chosen during the early phase characterized by low canopy cover, whereas shade-tolerant species are favored under conditions of high canopy cover. This approach is consistent with the ecological principle of aligning intercrop varieties with the available light resources throughout the rubber tree growth cycle.

### 2.2. Insect Sampling and Specimen Identification

Insect communities were surveyed using a combination of line transect netting, light trapping, and Malaise trapping to capture insects. (1) Line transect netting: Five parallel 100 m line transects were established in each plot, with ≥20 m between line transects. Investigators walked each transect at a slow and consistent pace, using sweep nets (handle length: 1.2 m; net bag diameter: 35 cm; bag depth: 80 cm; bag mesh size: 100 threads per inch) to conduct sampling. A “∞-shaped” sweeping technique was applied, with two sweeps performed per linear meter, targeting herbaceous vegetation and low shrubs within a 1.5 m wide corridor on either side of the transect. Each transect included a minimum of 200 sweeps (20 sweeps per 10 m segment over 10 segments). In addition, flying insects located within 3 m above the transect line were captured using netting method. (2) Light trapping: Nocturnal and phototactic insects were collected using a light-trapping tent, fitted with a 750 W high-pressure mercury vapor lamp mounted at the apex, during nights free of rainfall and strong winds. The trap was powered by a portable outdoor power source and positioned in relatively open areas within the central regions of each plot. Sampling was conducted between 20:00 and 03:00, with specimens collected promptly from the tent’s screening surface. (3) Malaise trapping: Flying insects were sampled continuously throughout the year using Malaise traps (dimensions: 1.7 m length, 1.2 m width, 1.8 m front height, 0.9 m rear height). The traps were set up in a tent-like formation, with each apex fitted with a collection bottle filled with 75% ethanol. To avoid capturing ground-dwelling arthropods, the bottles were suspended at a height of 1.8 m above the ground. All collection bottles were replaced concurrently across sampling plots according to a predefined schedule, ensuring temporal consistency among samples. The Malaise trap was set up in the central area of plot, positioned opposite to the light trap location, and placed in areas free of dense ground vegetation.

Insect sampling was conducted from April 2024 to March 2025, encompassing a complete dry- and rainy-season cycle on Hainan Island. Line transect netting and light trapping were carried out monthly during the mid-month period, while specimens from Malaise traps were collected on a predetermined monthly schedule (Table S1). Only adult insects with a body length of ≥2 mm were retained for analysis. The collected insect specimens were transported to the laboratory for taxonomic identification. Morphological characteristics served as the primary basis for identification, supplemented by reference to authoritative taxonomic literature, including *Fauna Sinica: Insecta* [30,31,32,33,34,35,36,37]. All specimens were identified to at least the family level; morphologically stable taxa were assigned to genus; and key or indicator groups were identified to species whenever feasible. Specimens with ambiguous morphological features were reviewed by taxonomic specialists, with genitalia dissection performed when necessary to confirm identity. For specimens that could not be confidently identified using morphological methods—particularly those representing abundant species—molecular identification was conducted via DNA barcoding.

In addition, each collected insect individual was assigned to a specific functional group based on mouthpart morphology and the primary feeding habits of the adult stage (Table S2). Insects were categorized into four core functional groups: herbivores, predators, detritivores, and omnivores [38,39]. Instances of functional ambiguity were resolved through a comprehensive literature review and consultation with domain experts. Insect functional structure was quantified based on the relative abundance and species composition of functional groups across different systems.

### 2.3. Environmental Factor Records

During the insect surveys, environmental variables including temperature, humidity, management intensity, canopy cover, vegetation cover, and flowering intensity were recorded concurrently with insect sampling. Temperature and humidity were measured using an RC-5 automatic recorder positioned 1.5 m above the ground along each transect at 1-hour intervals, expressed in degrees Celsius (°C) and percentage (%), respectively, and summarized as monthly averages. Management intensity was quantified through systematic recording of the frequency and resource inputs associated with management activities, including weeding and fertilization. Based on the integrative framework proposed by Blüthgen et al. [40], the eight plots were categorized into three intensity levels: low (annual routine fertilization accompanied by physical or ecological weed control), medium (an additional one to two management interventions per year attributable to agroforestry practices), and high (narrow-row planting, multiple fertilizations, weed control conducted at least twice annually, and frequent manual interventions). Canopy cover was estimated using the transect method by measuring the proportion of the canopy’s projected length along a 100 m transect. Vegetation cover and flowering intensity were estimated using a standardized quadrat-based visual method and recorded using the Braun–Blanquet cover scale [41] and the flowering phenology scale of Dafni et al. [42], respectively.

### 2.4. Data Analysis

All data integration and statistical analyses were conducted in R (v. 4.2.2; R Core Team, 2022), with two distinct datasets generated to align with analytical objectives: a “plot × month” dataset for multivariate analyses and a “plot × year” dataset for α-diversity assessment. To assess sampling sufficiency, rarefaction analysis was conducted using the vegan package: species richness was standardized to the minimum sample size (i.e., rarefied), and the resulting rarefied richness was compared against observed species richness; additionally, rarefaction curves were generated as a function of individual abundance. For the “plot × month” dataset, we computed species richness, individual abundance, and four alpha diversity indices (Shannon–Wiener diversity index, Simpson dominance index, Margalef richness index, and Pielou evenness index) [43] and visualized their monthly trajectories using line plots. To compare insect α-diversity metrics across agroforestry systems, annual mean values were computed from the “plot × year” dataset, and grouped bar plots were generated using ggplot2 for visualization.

Bray–Curtis dissimilarity matrices were computed from species abundance data (representing compositional structure) and functional group abundance data (representing functional structure), both derived from the “plot × month” dataset. Non-metric multidimensional scaling (NMDS) ordination was applied to visualize among-system variation in community structure, implemented via the metaMDS function in the vegan package. For functional structure, heatmap visualization—generated using the pheatmap package—depicted the cross-system distribution of mean species richness per functional group. To identify key environmental drivers, environmental vectors were fitted onto the NMDS ordination using the envfit function in vegan. Arrow direction and length reflect the strength and sign of the correlation between each environmental variable and the community ordination axes. Permutational multivariate analysis of variance (PERMANOVA) was subsequently applied using the adonis2 function to assess both the statistical significance and the proportion of variance explained by environmental predictors on community dissimilarity. To isolate the effects of the focal ecosystem types while accounting for temporal non-independence, the PERMANOVA model incorporated “month” as a stratification factor (strata) and “plantation age” as a continuous covariate—thereby controlling for temporal autocorrelation and adjusting for stand developmental stage, respectively. All significance tests were based on 9999 permutations to ensure statistical robustness.

## 3. Results

### 3.1. Insect Abundance, Species Richness and Diversity

A total of 94,483 insect specimens were collected across the eight sampling plots, belonging to 16 orders, 222 families, and 1560 species. The insect community was predominantly composed of Lepidoptera, Diptera, Hemiptera, and Coleoptera (Table 2). Lepidoptera displayed the highest species richness, encompassing 27 families, 375 genera, and 465 species, accounting for 29.8% of the total species recorded. Families within this order, particularly Noctuidae, Crambidae, and Erebidae, exhibited notable diversity. Diptera, represented by 45 families, 176 genera, and 236 species, contributed the largest number of individuals (*n* = 23,835), constituting 25.2% of all collected specimens, with Syrphidae, Muscidae, and Tachinidae identified as the dominant families. Coleoptera included 38 families, 218 genera, and 272 species, primarily comprising Chrysomelidae, Cerambycidae, and Scarabaeidae, and represented 17.4% of the total species. Hemiptera consisted of 36 families, 199 genera, and 230 species, totaling 14,349 individuals (15.2% of the overall abundance), with Cicadellidae, Miridae, and Pentatomidae being the most prevalent families. Collectively, Lepidoptera and Diptera accounted for 44.9% of all species and 47.8% of all individuals in the rubber-based agroforestry systems.

**Figure 3:**
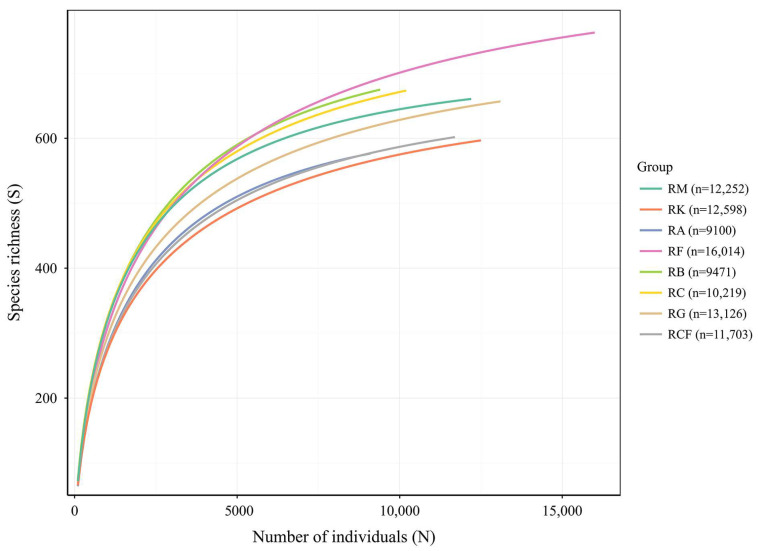
Individual-based rarefaction curves illustrating species richness of insect communities across rubber-based agroforestry systems. The x-axis shows the cumulative number of individuals sampled; the y-axis shows the estimated species richness. For each system, n denotes the total number of individuals (i.e., the observed sample size). RM: Rubber monoculture system; RK: Rubber–konjak agroforestry system; RA: Rubber–*Alpinia* agroforestry system; RF: Rubber–fig agroforestry system; RB: Rubber–banana agroforestry system; RC: Rubber–coconut agroforestry system; RG: Rubber–forage grass–black goat agroforestry system; RCF: Rubber–coconut–fig agroforestry system.

Based on raw observational data, the rubber–fig system supported the highest insect species richness (847 species) and abundance (16,014 individuals), whereas the rubber–*Alpinia* system recorded the lowest values for both metrics. Taxonomically, Lepidoptera and Diptera were the most species-rich and numerically dominant orders. Hemiptera exhibited the highest relative abundance in the rubber–fig system, while Hymenoptera consistently accounted for a substantial proportion across all systems (Table S3). To account for variation in sampling effort across agroforestry systems (individual counts ranged from 9100 to 16,014), we applied rarefaction to standardize all samples to the minimum observed sample size (9100 individuals). This approach mitigates bias arising from unequal sampling intensity. Following rarefaction, species richness estimates were recalculated (Figure 3): the rubber–banana system yielded the highest estimated richness (747.2 ± 0.23), surpassing the rubber–fig system’s raw (non-rarefied) richness value of 741.1 ± 0.79 (Table 3), indicating its greater potential taxonomic diversity. Sample coverage analysis indicated that all rubber-based agroforestry systems achieved high (>98%) and comparable coverage values, suggesting that species richness estimates are robust and that the sampling effort was sufficient to capture the majority of extant species within each community.

**Figure 4:**
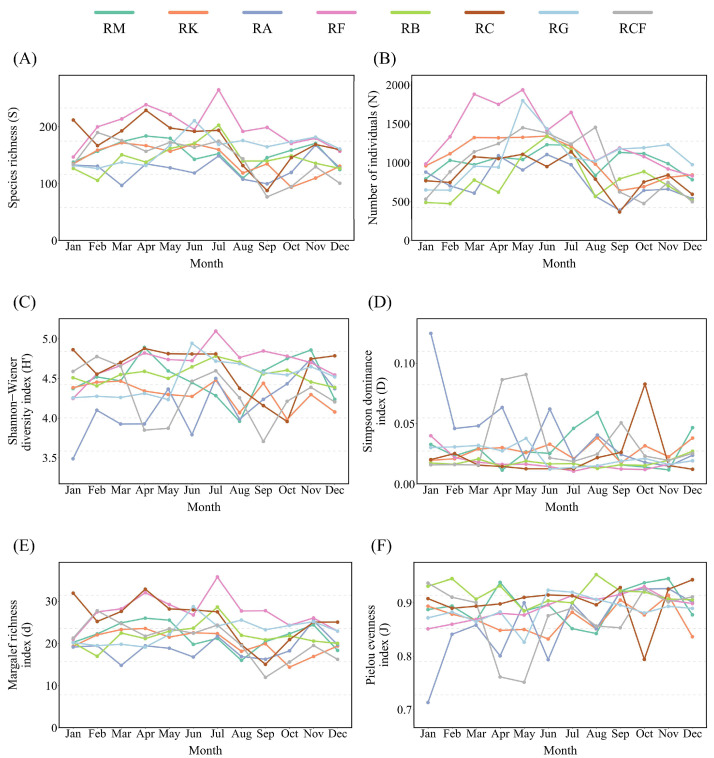
Temporal dynamics of insect community indices across rubber-based agroforestry systems: (**A**) Species richness; (**B**) Number of individuals; (**C**) Shannon–Wiener index; (**D**) Simpson dominance index; (**E**) Margalef richness index; (**F**) Pielou evenness index. RM: Rubber monoculture system; RK: Rubber–konjak agroforestry system; RA: Rubber–*Alpinia* agroforestry system; RF: Rubber–fig agroforestry system; RB: Rubber–banana agroforestry system; RC: Rubber–coconut agroforestry system; RG: Rubber–forage grass–black goat agroforestry system; RCF: Rubber–coconut–fig agroforestry system.

Monthly monitoring revealed a consistent seasonal pattern in insect alpha diversity across all rubber-based agroforestry systems, characterized by an initial increase followed by a subsequent decline in both species richness and total individual abundance (Figure 4). In the rubber–fig system, individual abundance peaked in March and May, whereas species richness peaked in July. In contrast, the rubber–*Alpinia* agroforestry system exhibited synchronous declines in both individual abundance and species richness in March and September. Three distinct seasonal turning points in insect alpha diversity were identified: (i) in January, the rubber–*Alpinia* agroforestry system reached annual minima in the Shannon–Wiener diversity index and Pielou evenness index, while the Simpson dominance index attained its annual maximum; (ii) in July, the rubber–fig and rubber–banana agroforestry systems simultaneously achieved annual maxima in species richness, the Shannon–Wiener diversity index, and the Margalef richness index; and (iii) in September, the rubber–coconut agroforestry system recorded annual minima in species richness, individual abundance, and the Margalef richness index; the rubber–coconut–fig agroforestry system showed annual minima in species richness, the Shannon–Wiener diversity index, and the Margalef richness index; and the rubber–*Alpinia* agroforestry system exhibited an annual minimum in individual abundance.

**Figure 5:**
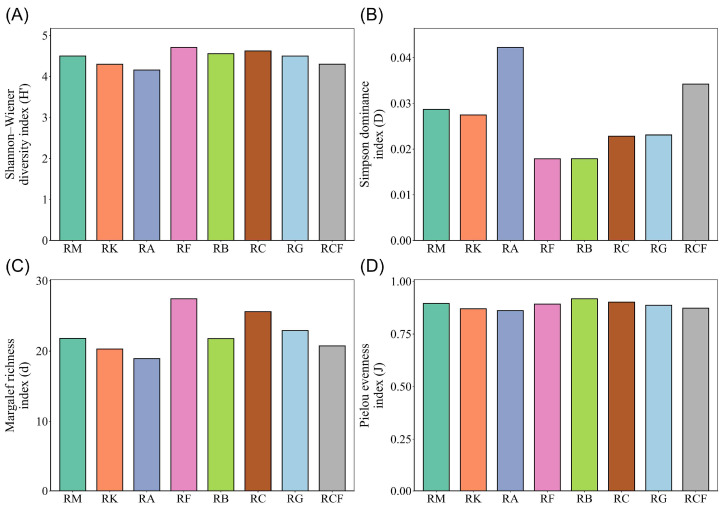
Annual mean insect community alpha diversity indices across rubber-based agroforestry systems. (**A**) Shannon–Wiener diversity index; (**B**) Simpson dominance index; (**C**) Margalef richness index; (**D**) Pielou evenness index. RM: Rubber monoculture system; RK: Rubber–konjak agroforestry system; RA: Rubber–*Alpinia* agroforestry system; RF: Rubber–fig agroforestry system; RB: Rubber–banana agroforestry system; RC: Rubber–coconut agroforestry system; RG: Rubber–forage grass–black goat agroforestry system; RCF: Rubber–coconut–fig agroforestry system.

A comparative analysis of insect alpha diversity across rubber-based agroforestry systems revealed the inter-system variation. Among the systems evaluated, the rubber–fig system exhibited the highest community complexity, as indicated by the highest annual mean Shannon–Wiener diversity index in all the systems. In contrast, the rubber–*Alpinia* system recorded the lowest values (Figure 5A). The Simpson dominance index remained comparatively low in both the rubber–fig and rubber–banana systems across all sampling periods (Figure 5B). Pronounced inter-system differences were observed in the Margalef richness index, with the rubber–fig agroforestry system exhibiting the highest annual mean value, driven predominantly by a marked peak in July (Figure 4E and Figure 5C). The rubber–coconut system also sustained relatively high richness throughout the year, whereas the rubber–*Alpinia*, rubber–coconut–fig, and rubber–konjak systems consistently yielded lower values (Figure 5C). Pielou’s evenness index was generally high across all systems (Figure 5D); however, temporal stability varied—the rubber–banana and rubber–fig systems demonstrated greater consistency, while the rubber–*Alpinia* and rubber–coconut–fig systems exhibited marked seasonal fluctuations, with minima occurring in January and April–May, respectively (Figure 4F).

Overall, the rubber–fig and rubber–banana agroforestry systems demonstrated superior performance in sustaining species diversity and stability, whereas the rubber monoculture system consistently maintained intermediate diversity levels across all assessed metrics. In contrast, the rubber–*Alpinia* system consistently ranked lowest across all evaluated indicators.

### 3.2. The Composition and Structure of Insect Communities

**Figure 6:**
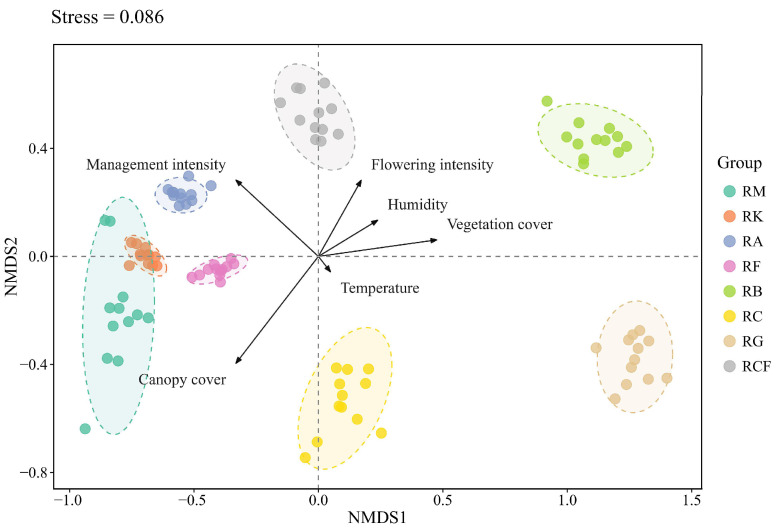
Non-metric multidimensional scaling (NMDS) ordination of insect species composition based on Bray–Curtis dissimilarities. Each point represents a plot-by-month sampling unit, colored by agroforestry system type; dashed ellipses indicate 95% confidence intervals around group centroids. Environmental vectors are fitted to the ordination space using envfit analysis, with arrow direction and length reflecting their correlation and strength of association with NMDS axes. The final NMDS stress value was 0.086, indicating an excellent fit to the two-dimensional solution. PERMANOVA—controlling for plantation age as a continuous covariate and sampling month as a stratum factor—confirmed that agroforestry system type had a highly significant effect on insect species composition (pseudo-R^2^ = 0.815, *p* = 0.001). RM: Rubber monoculture system; RK: Rubber–konjak agroforestry system; RA: Rubber–*Alpinia* agroforestry system; RF: Rubber–fig agroforestry system; RB: Rubber–banana agroforestry system; RC: Rubber–coconut agroforestry system; RG: Rubber–forage grass–black goat agroforestry system; RCF: Rubber–coconut–fig agroforestry system.

NMDS analysis revealed significant spatial heterogeneity in insect community composition and functional structure across rubber-based agroforestry systems, with distinct clustering patterns evident in both taxonomic and functional ordination space. PERMANOVA confirmed statistically significant differences among systems in insect species composition (*p* = 0.001) and functional trait composition (*p* = 0.001), whereas temporal variation within each system—assessed across seasons—was non-significant for both metrics (species composition: *p* = 0.417; functional traits: *p* = 0.565), indicating strong temporal stability of insect communities within individual agroforestry systems. NMDS ordination of insect species composition based on Bray–Curtis dissimilarity revealed distinct clustering patterns among rubber-based agroforestry systems (Figure 6). The rubber monoculture system exhibited high compositional similarity with the rubber–konjak and rubber–fig systems (Bray–Curtis dissimilarity values: 0.446 and 0.466, respectively), but was markedly differentiated from the rubber–forage grass–black goat and rubber–banana systems (0.796 and 0.785, respectively). The rubber–banana system displayed the highest degree of compositional uniqueness, with dissimilarity values exceeding 0.683 relative to all other systems except the rubber–forage grass–black goat system. In contrast, pairwise beta-diversity dissimilarities were comparatively low between the rubber–*Alpinia* and rubber–konjak systems (0.421) and between the rubber–fig and rubber–konjak systems (0.385) (Table S4).

**Figure 7:**
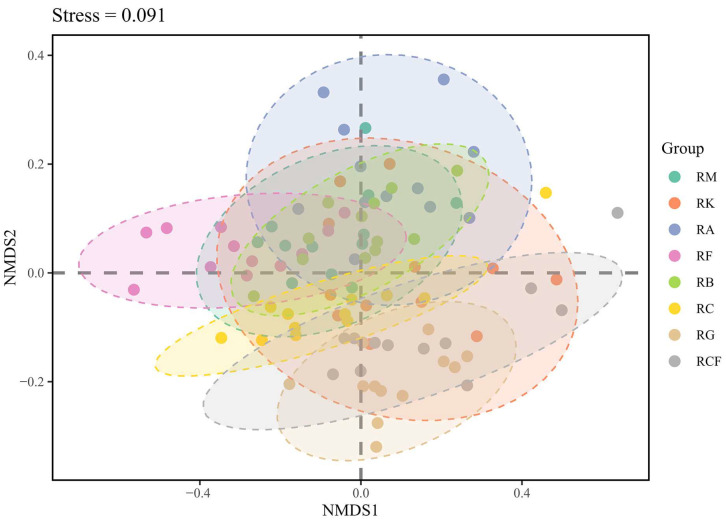
Non-metric multidimensional scaling (NMDS) ordination of insect functional structure based on Bray–Curtis dissimilarities. Each point represents a plot-by-month sampling unit, colored by agroforestry system type; dashed ellipses indicate 95% confidence intervals around group centroids. The final NMDS stress value was 0.091, indicating an excellent fit to the two-dimensional solution. PERMANOVA—controlling for plantation age as a continuous covariate and sampling month as a stratum factor—confirmed that agroforestry system type had a highly significant effect on insect functional structure (pseudo-R^2^ = 0.483, *p* = 0.001). RM: Rubber monoculture system; RK: Rubber–konjak agroforestry system; RA: Rubber–*Alpinia* agroforestry system; RF: Rubber–fig agroforestry system; RB: Rubber–banana agroforestry system; RC: Rubber–coconut agroforestry system; RG: Rubber–forage grass–black goat agroforestry system; RCF: Rubber–coconut–fig agroforestry system.

Based on feeding habits, a total of 1560 insect species were assigned to four trophic functional groups: herbivores (*n* = 1024; 65.6%), predators (*n* = 301; 19.3%), omnivores (*n* = 150; 9.6%), and detritivores (*n* = 85; 5.5%). Insect functional structure exhibited substantial overlap among the rubber-based agroforestry systems (Figure 7). Although the rubber monoculture and rubber–*Alpinia* systems exhibited marked dissimilarity in species composition (Bray–Curtis dissimilarity = 0.506) (Table S4), they showed strong functional convergence (functional dissimilarity = 0.104) (Table S5). Nevertheless, the rubber monoculture system remained distinctly separated from the rubber–forage grass–black goat system in both insect community composition and functional ordination space.

**Figure 8:**
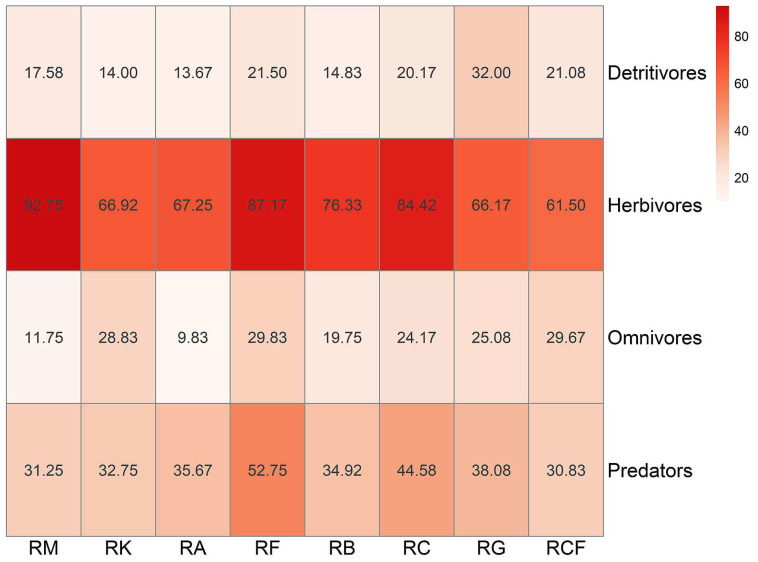
Heatmap illustrating the spatial distribution of insect functional groups across rubber-based agroforestry systems. Columns denote distinct habitat types, while rows correspond to insect functional groups. A red color gradient—ranging from light (low species richness) to dark (high species richness)—encodes mean species richness values. Numeric annotations within each cell indicate the corresponding mean species richness per functional group–habitat combination. RM: Rubber monoculture system; RK: Rubber–konjak agroforestry system; RA: Rubber–*Alpinia* agroforestry system; RF: Rubber–fig agroforestry system; RB: Rubber–banana agroforestry system; RC: Rubber–coconut agroforestry system; RG: Rubber–forage grass–black goat agroforestry system; RCF: Rubber–coconut–fig agroforestry system.

Heatmap visualization further revealed system-specific patterns of species richness across trophic functional groups (Figure 8). Herbivores exhibited the highest species richness in all systems, with maxima observed in the rubber monoculture (92.75 species per plot-month) and rubber–fig systems (87.17). Predators attained their highest species richness in the rubber–fig system (52.75), followed by the rubber–coconut system (44.58), but were substantially lower in the rubber–coconut–fig and rubber monoculture systems. Detritivores showed moderate inter-system variation in species richness, peaking in the rubber–forage grass–black goat system (32.00) and reaching their minimum in the rubber–*Alpinia* system (13.67). Omnivores displayed the lowest species richness in the rubber–*Alpinia* system (9.83), yet maintained comparatively higher values in the rubber–fig, rubber–coconut–fig, and rubber–konjak systems. Collectively, the rubber–fig system supported consistently high species richness across multiple functional groups.

### 3.3. Associations Between Insect Communities and Environmental Predictors

NMDS coupled with envfit analysis revealed that insect communities across rubber-based agroforestry systems were significantly associated with distinct sets of environmental predictors, including temperature, humidity, vegetation cover, flowering intensity, canopy cover, and management intensity (Figure 6). Distinct environmental predictors exhibited differential associations with insect community patterns. Canopy cover (R^2^ = 14.65%, *p* < 0.001) and management intensity (R^2^ = 11.54%, *p* < 0.001) emerged as the two strongest correlates (Table 4). The management intensity vector aligned closely with the positive NMDS2 and negative NMDS1 axes, indicating a strong positive association with the rubber–*Alpinia* and rubber–coconut–fig systems, habitats characterized by elevated anthropogenic disturbance. In contrast, the canopy cover vector pointed toward the negative direction of both NMDS axes, reflecting a robust association with the rubber monoculture system habitats, thereby distinguishing these closed-canopy systems from the more open-canopy rubber–banana system habitats. Flowering intensity (R^2^ = 9.88%, *p* < 0.001) ranked as the third in explanatory power; its vector projected upward along NMDS2, showing strong positive correlation with the rubber–coconut–fig system. Vegetation cover (R^2^ = 8.07%, *p* < 0.001) correlated positively with NMDS1, corresponding to the rubber–forage grass–black goat and rubber–banana systems. Humidity exhibited a significant positive association with insect communities in the rubber–banana system located along the positive NMDS1 axis, whereas temperature showed a significant positive association specifically with the rubber–coconut system (Figure 6). Collectively, canopy cover and management intensity constituted the primary environmental gradients structuring spatial variation in insect community composition across rubber-based agroforestry systems.

## 4. Discussion

### 4.1. Distribution Patterns and Drivers of Insect Communities Across Rubber-Based Agroforestry Systems

Insects, being highly diverse and closely associated with vegetation, are sensitive indicators of habitat changes and, therefore, serve as reliable proxies for assessing ecosystem health [44,45]. Our comparative analysis across rubber-based agroforestry systems demonstrates that planting patterns play a pivotal role in shaping insect community composition and structure [46]. This influence is primarily mediated by variations in vegetation architecture—including plant species composition, vertical stratification, and canopy cover—and the resulting understory microclimatic conditions (such as temperature and humidity), which jointly affect habitat heterogeneity and resource availability for insect communities [47,48,49]. Structurally more complex agroforestry systems including the rubber–fig and rubber–banana systems consistently supported higher insect species richness and α-diversity. This pattern is likely attributable to their provision of diversified ecological niches and spatially heterogeneous microhabitats, thereby facilitating coexistence among insect taxa exhibiting divergent life-history strategies and trophic specializations [50,51,52,53,54,55]. In contrast, the rubber monoculture system, characterized by simplified vertical and horizontal vegetation structure, exhibited relatively lower species richness and α-diversity; these findings corroborate a substantial body of evidence demonstrating that monoculture practices systematically reduce arthropod biodiversity through habitat homogenization and resource limitation [56,57].

However, increased structural complexity alone does not invariably enhance insect diversity. In the rubber–coconut–fig system, the distinctive canopy architecture likely modifies the understory microclimate, indirectly suppressing understory plant productivity and flowering phenology—thereby constraining insect species composition [58]. Moreover, in the rubber–*Alpinia* and rubber–konjak systems, interactions among co-occurring plant species may result in competitive shading or resource overlap, which can reduce the availability of suitable microhabitats [59]. Therefore, the ecological benefits of diversification are contingent upon the functional arrangement and species complementarity within the system. Moderate levels of shading and resource heterogeneity (such as the mixed herbaceous–litter structure generated by grazing in the rubber–forage grass–black goat system) may contribute to enhanced habitat quality. In contrast, excessive canopy cover may inhibit understory development and offset potential biodiversity gains [60,61].

At the trophic level, marked differences in functional group composition reflect how insect assemblages respond to resource availability and disturbance regimes. Structurally complex and heterogeneous systems promote distinct species pools and differentiated functional responses [55,62]. In the rubber monoculture system, herbivores were strongly dominant, likely due to the simplified vegetation structure supporting fewer natural enemies, which allows herbivore populations to proliferate unchecked [63]. In contrast, the rubber–forage grass–black goat system benefits from moderate grazing, which not only concentrates detritivorous insects through the input of feces and residual plant material but also provides essential resources for detritivorous and herbivorous intermediate prey. This process indirectly establishes a “prey pool” that sustains predatory insect populations [64]. Grazing-induced disturbances further generate spatially heterogeneous microhabitats—characterized by a mosaic of low herbaceous vegetation and surface litter—enhancing foraging opportunities and refuge availability for predators, thereby promoting the aggregation and persistence of natural enemy communities [60]. In the rubber–konjak system, intercropping with konjac increases soil organic matter content and enhances microhabitat heterogeneity, resulting in greater abundance of omnivorous insects. This pattern highlights the strong linkage between plant functional traits and the trophic structure of insect communities [65]. The rubber–fig system exhibited a more balanced trophic structure, attributable to vertical structural complementarity between the fig shrub layer and the overstory rubber canopy—a configuration that markedly enhanced habitat heterogeneity [66]. This multi-layered architecture likely provided critical microhabitat refugia and foraging resources for natural enemies, including carabid beetles and parasitoid wasps, thereby reinforcing top-down regulation of herbivore populations.

A notable finding of this study is the decoupling of taxonomic composition from functional structure. This pattern reflects substantial functional redundancy within the insect communities: herbivorous and predatory species in one rubber-based agroforestry system can be replaced by phylogenetically distinct taxa that perform equivalent trophic functions. Such redundancy likely contributes to the temporal stability observed across rubber-based agroforestry systems and supports the persistence of core ecosystem functions—including herbivory and predation—despite substantial species turnover [67]. This functional buffering capacity represents a key ecological attribute that enhances community resilience to environmental perturbations.

### 4.2. Relationship Between Insect Communities and Environmental Predictors

In rubber-based agroforestry systems, insect species assembly is governed by multidimensional environmental gradients, with canopy cover and management intensity emerging as the predominant drivers. Canopy cover, a key structural attribute of vegetation, exerts ecologically complex effects. Moderate canopy cover, as exemplified in the rubber–banana and rubber–fig systems, enhances understory light heterogeneity and microclimatic variability, thereby supporting higher insect species richness and α-diversity. In contrast, high canopy cover in the rubber monoculture system restricts understory resource availability, likely resulting in reduced habitat structural complexity and a depauperate community structure [68]. Concurrently, high-intensity anthropogenic disturbances, including frequent latex tapping, mechanical weeding, and synthetic fertilization, are associated with habitat homogenization and vegetation structural simplification, collectively diminishing habitat suitability for ecologically specialized functional groups (e.g., parasitoid wasps and oligophagous herbivores) [69,70]. Notably, in structurally complex systems such as the rubber–forage grass–black goat system, the adverse effects of management intensity on insect assemblages are markedly attenuated indicating that diversified plant architecture can effectively buffer against human-induced habitat degradation [62].

Flowering intensity emerged as another significant positive predictor of insect species composition. Flowering plants supply pollen and nectar—key floral resources that not only directly sustain pollinator populations but also, via bottom-up regulation, indirectly support higher-trophic-level consumers such as predators [71,72]. Both vegetation cover and humidity exert significant and consistent influences on insect assemblages across compositional and functional dimensions. Higher humidity modulates understory microclimatic conditions and soil moisture regimes, thereby promoting the assembly of structurally complex and temporally stable insect communities [73,74]. Vegetation cover enhances community stability by providing spatially heterogeneous microhabitats and diversified trophic resources [75,76]. In contrast, temperature exerts a comparatively weaker—yet ecologically consequential—effect. Elevated temperatures may intensify desiccation stress, thereby suppressing insect activity, development rates, and local persistence. Functionally, this thermal effect is likely to be partially mitigated by behavioral thermoregulation and physiological plasticity, thereby attenuating selective pressure on functional guild composition [77].

Overall, insect community organization in rubber plantations is shaped not by a single dominant driver but by the interplay among canopy cover, management intensity, and resource availability—particularly vegetation cover and flowering intensity. Our findings demonstrate that the structural complexity of rubber-based agroforestry systems can effectively buffer against the adverse effects of intensive management, whereas targeted provisioning of floral resources is essential for sustaining key ecosystem functions. These results provide an evidence-based foundation for refining rubber-based agroforestry design: specifically, by minimizing anthropogenic disturbance while integrating structurally heterogeneous vegetation and complementary floral resource phenologies to concurrently advance biodiversity conservation and ecosystem functionality.

The forest canopy’s three-dimensional architecture fosters insect assemblage diversity by generating heterogeneous microhabitats, establishing vertical climatic gradients, and provisioning trophic resources and predation refuge. However, this structural complexity poses significant methodological constraints for insect sampling: active techniques systematically under-sample cryptic microhabitats, particularly within the upper canopy stratum and branch interiors, whereas passive trapping efficacy is strongly modulated by canopy-mediated effects on insect flight behavior and semiochemical dispersion. In rubber-based agroforestry systems characterized by low intercropped vegetation cover, the rubber tree canopy constitutes the predominant structural layer of the canopy across all study plots; consequently, targeted sampling of rubber tree canopies—including the deployment of Malaise traps within the canopy—was not conducted, representing a limitation of this study. To ensure robust characterization of insect assemblages in future research, a comprehensive sampling strategy is required that explicitly incorporates vertical stratification (canopy versus understory), methodological complementarity (active versus passive sampling techniques), and standardized deployment protocols across habitat strata.

## 5. Conclusions

Canopy cover and management practices are critical determinants of insect biodiversity and ecosystem function in agroforestry systems. This study systematically evaluated eight rubber-based agroforestry systems in Hainan and found that the rubber–fig and rubber–banana agroforestry systems most effectively maintained insect species richness, diversity, and community stability, demonstrating strong ecological sustainability. In contrast, the rubber–*Alpinia* system exhibited excessive canopy cover, which limited understory light availability and constrained the ecological functions of insect communities. At the functional level, composite systems supported a more complete trophic structure. Moderate grazing in the rubber–forage grass–black goat agroforestry system enhanced the aggregation of predatory and detritivorous insects by increasing litter and fecal inputs and generating heterogeneous microhabitats, whereas intercropping with konjac in the agroforestry system attracted more omnivorous insects, reflecting the influence of plant functional traits on insect feeding guilds. Environmental analyses indicated that vegetation cover and flowering intensity were key drivers of insect communities, while canopy cover and intensive human management were the primary factors. Based on these findings, we recommend prioritizing ecologically beneficial composite models, such as the rubber–fig and rubber–banana (specific to the rubber-based agroforestry system during the low canopy cover stage) agroforestry systems, to enhance ecological functions by maintaining understory vegetation and extending the availability of nectar and pollen resources. For the rubber–coconut–fig agroforestry system, canopy structure optimization is advised, while moderate grazing in the rubber–forage grass–black goat agroforestry system can serve as an effective habitat management strategy.

Overall, this study demonstrates that the ecological benefits of rubber-based agroforestry systems are governed not only by crop diversity but also by the complexity of vegetation structure, resource availability, and management intensity. These insights provide a scientific foundation and practical guidance for the sustainable management of tropical rubber agroforestry, contributing to the transformation of conventional rubber cultivation toward ecologically resilient and biodiversity-friendly practices.

## Figures and Tables

**Table 1:** Descriptive characteristics of the experimental plots.

Plot No.	Intercropping System	Rubber Plantation Age (Years)	Rubber Planting Patterns	Canopy Cover (%)	Coverage (%)	Rubber Tree Management Measures	Manual Management Practices for Intercropped Crops	Understory Vegetation
I	Rubber–bannana (*Musa nana*) (RB)	2	(20 + 4) m × 2 m	0~5	>55%	No tapping; Narrow-row fertilization, grass pressing twice a year	Manual fertilization and weed control (~30 times/year)	Wide rows: mainly banana; Narrow rows: few sun-loving weeds
II	Rubber–coconut (*Cocos nucifera*) (RC)	8	(20 + 4) m × 2 m	30~40	>50%	Tapped every 3 days for one year; Narrow-row fertilization, grass pressing twice a year	Leaf thinning once a year	Wide rows: coconut/sun-loving weeds; Narrow rows: few shade-tolerant weeds
III	Rubber–coconut (*Cocos nucifera*)–fig (*Ficus hirta*) (RCF)	7	(20 + 4) m × 2 m	30~40	>65%	No tapping; Regular fertilization once a year	*Cocos nucifera* pruning once a year; *Ficus hirta* fertilization and weed control twice a year	Wide rows: coconut/fig/few sun-loving weeds; Narrow rows: few shade-tolerant weeds
IV	Rubber–fig (*Ficus hirta*) (RF)	9	(20 + 4) m × 2 m	35~40	>50%	Tapped every 3 days for 2 years; Regular weed control and annual fertilization	Manual fertilization and weed control twice a year	Wide rows: mainly fig and few sun-loving weeds; Narrow rows: few shade-tolerant weeds
V	Rubber–forage grass (*Brachiaria eruciformis*)–black goat (RG)	6	7 m × 3 m	50~65	>55%	No tapping; Regular fertilization once a year	Rotational grazing 3 times per year, supplementary fertilization in dry season	Forage grass dominant
VI	Rubber– konjak (*Amorphophallus konjac*) (RK)	10	7 m × 3 m	65~75	>45%	Tapped every 3 days for 3 years; Regular weed control and annual fertilization	Mulching for weed control, chemical disease prevention	Primarily konjak and few shade-tolerant herbaceous weeds
VII	Rubber–*Alpinia* (*Alpinia oxyphylla*) (RA)	10	7 m × 3 m	78~85	>65%	Tapped every 3 days for 3 years; Regular weed control and annual fertilization	Pruning and leaf harvesting once a year, manual fertilization and annual weed control	*Alpinia oxyphylla* dominant, few shade-tolerant weeds
VIII	Rubber monoculture (RM)	8	7 m × 3 m	78~85	>55%	Tapped every 3 days for one year; Regular weed control and annual fertilization	None	Primarily shade-tolerant herbaceous weeds

**Table 2:** Insect community structure in rubber-based agroforestry systems.

Order	No. of Families (%)	No. of Genera (%)	No. of Species (%)	No. of Individuals (%)
Hemiptera	36 (16.2%)	199 (16.3%)	230 (14.7%)	14,349 (15.2%)
Diptera	45 (20.3%)	176 (14.4%)	236 (15.1%)	23,835 (25.2%)
Psocodea	2 (0.9%)	2 (0.2%)	2 (0.1%)	105 (0.1%)
Trichoptera	4 (1.8%)	9 (0.7%)	10 (0.6%)	193 (0.2%)
Orthoptera	11 (5.0%)	51 (4.2%)	81 (5.2%)	10,861 (11.5%)
Neuroptera	4 (1.8%)	14 (1.1%)	18 (1.2%)	463 (0.5%)
Hymenoptera	29 (13.1%)	132(11%)	191 (12.2%)	12,299 (13.0%)
Ephemeroptera	1 (0.5%)	1 (0.1%)	1 (0.1%)	163 (0.2%)
Blattodea	7 (3.2%)	14 (1.1%)	16 (1.0%)	625 (0.7%)
Odonata	5 (2.3%)	11 (0.9%)	15 (1.0%)	90 (0.1%)
Mantodea	5 (2.3%)	8 (0.7%)	10 (0.6%)	58 (0.1%)
Dermaptera	4 (1.8%)	6 (0.5%)	6 (0.4%)	135 (0.1%)
Coleoptera	38 (17.1%)	218 (17.9%)	272 (17.4%)	9677 (10.2%)
Lepidoptera	27 (12.2%)	375 (30.7%)	465 (29.8%)	21,328 (22.6%)
Phasmatodea	2 (0.9%)	2 (0.2%)	3 (0.2%)	289 (0.3%)
Megaloptera	2 (0.9%)	2 (0.2%)	4 (0.3%)	13 (0.001%)
Total	222	1220	1560	94,483

**Table 3:** Species richness of insect communities across rubber-based agroforestry systems.

Group	Observed Species Richness	Rarefied Species Richness (±SE)	Sample Coverage (%)
RM	708	680.2 ± 0.43	99.37
RK	662	617.2 ± 0.59	99.05
RA	639	639.0 ± 0.00	98.79
RF	847	741.1 ± 0.79	98.95
RB	753	747.2 ± 0.23	98.53
RC	756	736.3 ± 0.41	98.38
RG	730	673.3 ± 0.60	98.86
RCF	667	632.6 ± 0.52	98.94

Note: Rarefied species richness (±SE) indicates species richness standardized to the minimum sample size, with standard error. Sample coverage (%) denotes the estimated proportion of individuals in the community that are represented by at least one observed specimen in the sample, as calculated using Good’s coverage estimator. RM: Rubber monoculture system; RK: Rubber–konjak agroforestry system; RA: Rubber–*Alpinia* agroforestry system; RF: Rubber–fig agroforestry system; RB: Rubber–banana agroforestry system; RC: Rubber–coconut agroforestry system; RG: Rubber–forage grass–black goat agroforestry system; RCF: Rubber–coconut–fig agroforestry system.

**Table 4:** Permutational multivariate analysis of variance (PERMANOVA) results evaluating the effects of environmental predictors on insect communities, with plantation age treated as a continuous covariate and sampling month specified as a stratum factor.

Environmental Predictors	Explained Variance (%)	F-Value	*p*-Value
Canopy cover	14.65	23.85	<0.001 ***
Management intensity	11.54	18.69	<0.001 ***
Flowering intensity	9.88	16.30	<0.001 ***
Vegetation cover	8.07	13.07	<0.001 ***
Humidity	5.42	8.78	<0.001 ***
Temperature	3.72	6.06	<0.001 ***

Note: PERMANOVA was conducted using Bray–Curtis dissimilarity matrices. Plantation age was included as a continuous covariate and sampling month as a stratum factor to control for temporal and developmental confounding effects. Explained variance (%) corresponds to the pseudo-R^2^ statistic (permutational R^2^) for each term in the model. Significance was assessed via 9999 unrestricted permutations of the residuals under the reduced model. *** *p* < 0.001.

## Data Availability

The specimens were deposited in the Forest Insect Laboratory, School of Tropical Agriculture and Forestry, Hainan University. All raw data are described in the Supplementary Materials File.

## Supplementary Materials

The following supporting information can be downloaded at https://www.mdpi.com/article/10.3390/insects17020195/s1. Table S1: Detailed schedule of insect sampling campaigns conducted from April 2024 to March 2025; Table S2: List of the collected insects as well as the functional groups in rubber-based agroforestry systems; Table S3: Diversity and composition of insect communities across rubber-based agroforestry systems; Table S4: Bray–Curtis dissimilarity matrix for insect community composition across rubber-based agroforestry systems; Table S5: Bray–Curtis dissimilarity matrix for insect functional structure across rubber-based agroforestry systems.
