# Supplementary material for: Microclimatic and Anthropogenic Drivers of Insect Biodiversity in Rubber-Based Agroforestry Systems
Source: Insects. 2026 Feb 12;17(2):195. doi: 10.3390/insects17020195 (PMC12942340; doi:10.3390/insects17020195)
Supplement: Supplementary file 1 [file insects-17-00195-s001.zip › Supplementary Materials.pdf]

Supplementary Materials

Table S1. Detailed schedule of insect sampling campaigns conducted from April 2024 to March 2025.

Period (Year-Month)	Instantaneous Sampling (Sweep Netting & Light Trapping)	Collection time per bottle (Malaise trapping)
Apr-24	**(SN):** 15 April 2024<br>**(LT):** 14 April 2024 – 17 April 2024	**(MT):** 1 April 2024 – 30 April 2024
May-24	**(SN):** 15 May 2024<br>**(LT):** 14 May 2024 – 17May 2024	**(MT):** 1 May 2024 – 31 May 2024
Jun-24	**(SN):** 14 June 2024<br>**(LT):** 14 June 2024, 16 June 2024 – 18 June 2024	**(MT):** 1 June 2024 – 30 June 2024
Jul-24	**(SN):** 15 July 2024<br>**(LT):** 14 July 2024 – 17 July 2024	**(MT):** 1 July 2024 – 31 July 2024
Aug-24	**(SN):** 15 August 2024<br>**(LT):** 15 August 2024 – 18 August 2024	**(MT):** 1 August 2024 – 31 August 2024
Sep-24	**(SN):** 15 September 2024<br>**(LT):** 14 September 2024 – 17 September 2024	**(MT):** 1 September 2024 – 30 September 2024
Oct-24	**(SN):** 15 October 2024<br>**(LT):** 14 October 2024 – 17 October 2024	**(MT):** 1 October 2024 – 31 October 2024
Nov-24	**(SN):** 15 November 2024<br>**(LT):** 14 November 2024 – 17 November 2024	**(MT):** 1 November 2024 – 30 November 2024
Dec-24	**(SN):** 15 December 2024<br>**(LT):** 14 December 2024 – 17 December 2024	**(MT):** 1 December 2024 – 31 December 2024
Jan-25	**(SN):** 15 January 2025<br>**(LT):** 14 January 2025 – 17 January 2025	**(MT):** 1 January 2025 – 31 January 2025
Feb-25	**(SN):** 15 February 2025<br>**(LT):** 14 February 2025 – 17 February 2025	**(MT):** 1 February 2025 – 28 February 2025

Mar-2025	**(SN):** 15 March 2025<br>**(LT):** 14 March 2025 – 17 March 2025	**(MT):** 1 March 2025 – 31 March 2025

Note: The table presents the sampling periods for three methods across different months: sweep netting (SN, instantaneous), malaise trapping (MT, continuous monthly), and light trapping (LT, intermittent). Light trapping was conducted on discontinuous dates due to weather-dependent constraints (e.g., requirement for calm and rainless nights), with sampling efforts concentrated in the mid-month period to standardize sampling intensity and optimize collection conditions.

Table S2. List of the collected insects as well as the functional groups in rubber-based agroforestry systems (This table is provided as a standalone Excel file owing to its substantial data volume).

Table S3. Diversity and composition of insect communities across rubber-based agroforestry systems.

Order	RM		RK		RA		RF		RB		RC		RG		RCF	
	Sp.	Ind.	Sp.	Ind.	Sp.	Ind.	Sp.	Ind.	Sp.	Ind.	Sp.	Ind.	Sp.	Ind.	Sp.	Ind.
Hemiptera	70	1620	75	1921	56	860	102	2782	80	2145	85	1861	91	1789	80	1371
Diptera	93	2472	76	2940	74	2235	102	4183	111	2174	67	1814	110	4701	82	3316
Psocodea	2	26	2	26	-	-	1	21	1	25	1	7	-	-	-	-
Trichoptera	5	38	4	43	2	14	5	28	4	39	3	8	4	11	5	12
Orthoptera	48	1545	35	2196	41	1512	47	2260	42	534	64	1637	45	575	43	602
Neuroptera	7	56	9	173	7	33	10	106	8	28	11	35	1	3	7	29
Hymenoptera	95	1526	67	1745	64	636	98	2144	78	1060	70	1572	89	1708	70	1908
Ephemeroptera	1	33	1	14	1	9	1	85	1	7	1	10	1	4	1	1
Blattodea	7	120	7	72	6	50	6	142	7	75	4	20	5	110	5	36
Odonata	3	5	5	7	3	6	5	8	6	22	3	3	6	34	3	5
Mantodea	4	9	2	2	2	6	3	8	2	6	5	18	3	7	1	2
Dermaptera	2	25	1	3	1	4	1	13	4	23	1	1	3	60	3	6
Coleoptera	82	1530	90	936	78	815	110	1748	97	1166	82	726	79	1803	90	953
Lepidoptera	287	3155	288	2520	303	2915	353	2360	310	2163	355	2448	293	2321	275	3446
Phasmatodea	2	92	-	-	-	-	2	124	-	-	2	57	-	-	2	16
Megaloptera	-	-	-	-	1	5	1	2	2	4	2	2	-	-	-	-
Total	708	12,252	662	12,598	639	9100	847	16,014	753	9471	756	10,219	730	13,126	667	11,703

Note：RM: Rubber monoculture system, RK: Rubber-konjak agroforestry system, RA: Rubber-*Alpinia* agroforestry system, RF: Rubber-fig agroforestry system, RB: Rubber-banana agroforestry system, RC: Rubber-coconut agroforestry system, RG: Rubber-forage grass-black goat agroforestry system, RCF: Rubber-coconut-fig agroforestry system.

Table S4. Bray-Curtis dissimilarity matrix for insect community composition across rubber-based agroforestry systems.

Group	RA	RB	RC	RCF	RF	RG	RK	RM
RA	0	0.764	0.665	0.535	0.447	0.781	0.421	0.506
RB		0	0.684	0.683	0.723	0.596	0.802	0.785
RC			0	0.667	0.551	0.677	0.640	0.645
RCF				0	0.576	0.711	0.631	0.655
RF					0	0.754	0.385	0.466
RG						0	0.804	0.796
RK							0	0.446
RM								0

Note: Analyses were based on the Bray–Curtis dissimilarity matrix. Values range from 0 (identical composition) to 1 (no shared species), reflecting the degree of compositional dissimilarity between agroforestry system pairs. PERMANOVA—accounting for plantation age as a continuous covariate and sampling month as a stratum factor—confirmed a highly significant effect of agroforestry system on insect community composition ($R^2 = 0.815$, $P < 0.001$). RA: Rubber-*Alpinia* agroforestry system; RB: Rubber-banana agroforestry system; RC: Rubber-coconut agroforestry system; RCF: Rubber-coconut-fig agroforestry system; RF: Rubber-fig agroforestry system; RG: Rubber-forage grass-black goat agroforestry system; RK: Rubber-konjak agroforestry system; RM: Rubber monoculture system.

Table S5. Bray-Curtis dissimilarity matrix for insect functional structure across rubber-based agroforestry systems.

Group	RA	RB	RC	RCF	RF	RG	RK	RM
RA	0	0.086	0.175	0.171	0.216	0.209	0.137	0.104
RB		0	0.121	0.142	0.162	0.175	0.091	0.077
RC			0	0.128	0.141	0.139	0.102	0.106
RCF				0	0.238	0.083	0.088	0.153
RF					0	0.246	0.190	0.145
RG						0	0.125	0.216
RK							0	0.104
RM								0

Note: Analyses were based on the Bray–Curtis dissimilarity matrix. Values range from 0 (identical composition) to 1 (no shared species), reflecting the degree of compositional dissimilarity between agroforestry system pairs. PERMANOVA—accounting for plantation age as a continuous covariate and sampling month as a stratum factor—confirmed a highly significant effect of agroforestry system on insect functional structure ($R^2 = 0.483$, $P < 0.001$). RA: Rubber-*Alpinia* agroforestry system; RB: Rubber-banana agroforestry system; RC: Rubber-coconut

agroforestry system; RCF: Rubber-coconut-fig agroforestry system; RF: Rubber-fig agroforestry system; RG: Rubber-forage grass-black goat agroforestry system; RK: Rubber-konjak agroforestry system; RM: Rubber monoculture system.
